# Multiphoton microscopy: a personal historical review, with some future predictions

**DOI:** 10.1117/1.JBO.25.1.014511

**Published:** 2020-01-22

**Authors:** Colin J. R. Sheppard

**Affiliations:** a Istituto Italiano di Tecnologia, Department of Nanophysics, Genova, Italy; b University of Wollongong, School of Chemistry, Wollongong, New South Wales, Australia

**Keywords:** multiphoton microscopy, two-photon fluorescence, second-harmonic generation, focal modulation microscopy, image scanning microscopy

## Abstract

The historical development of multiphoton microscopy is described, starting with a review of two-photon absorption, and including two- and three-photon fluorescence microscopies, and second- and third-harmonic generation microscopies. The effects of pulse length on signal strength and breakdown are considered. Different contrast mechanisms, including use of nanoparticles, are discussed. Two new promising techniques that can be applied to multiphoton microscopy are described.

## 1 Two-Photon Absorption

Perhaps the first mention of two-photon absorption was by Dirac,^1^ who said:

“These terms correspond to processes in which two light-quanta are emitted or absorbed simultaneously, and cannot arise in a light-quantum theory in which there are no forces between the light quanta. The effects of these terms will be found to be negligible, so that this disagreement with the light-quantum theory is not serious.”

So he seems to say that two-photon absorption is not a physical effect but an artifact of his theory, and that as this effect is small, we can neglect it. Then in the first edition of his book *The Principles of Quantum Mechanics*, he says, on p. 231:^2^

“The extra terms in (22) would correspond to transitions in which two photons are simultaneously absorbed or emitted and the possibility of such transitions requires a more complicated interaction energy than that assumed in (9). The physical effects of these terms are, however, small and unimportant, and so we shall neglect them.”

So here he again says that the two-photon effects are small and neglectable but would be physically described by a more complicated theory. Then, much later, in the fourth edition (1958),^3^ on p. 239:

“Similarly for a more general radiative process in which two or more photons are simultaneously emitted or absorbed, the probability is proportional to a factor $I_{\nu 1}$ for each absorbed photon and $I_{\nu 1}+h\nu^{3}/c^{2}$ for each emitted photon. Thus the process is stimulated by incident radiation in the same direction and with the same frequency and polarization as any of the emitted photons.”

And on p. 244:

“The matrix elements referring to simultaneous absorption or emission of two photons may be written down in the same way, but they lead to physical effects too small to be of practical importance.”

Dirac also introduced the quantum theory for Rayleigh scattering and Raman scattering.^4^

The full theory of two-photon absorption was developed by Göppert-Mayer.^5^^–^^8^ She received her PhD in 1930, whereas Dirac had received his in 1926. It is interesting to note that Dirac spent February to June 1927 and June 1928 in Göttingen visiting Max Born, Göppert-Meyer’s supervisor.^9^ During the first visit, he submitted two papers, “The quantum theory of emission and absorption of radiation,” which introduces the quantum theory of creation and annihilation of photons, and “The quantum theory of dispersion.”^1^^,^^4^

The influence of Dirac on Göppert-Mayer’s work was investigated by Masters:^10^

“The significance of this careful reconstruction of her Göttingen dissertation, together with a thorough comparison of the two papers that Dirac published in 1927, demonstrates that Göppert not only used and cited Dirac’s papers, but the extent to which she incorporated theoretical techniques from those two papers is significant. Previously, this incorporation of Dirac’s work into her Göttingen dissertation has either not been described or has been ignored in the literature on the history of quantum mechanics.”

Masters also discusses earlier related work and its influence on Göppert-Mayer.^10^

Various different nonlinear processes were observed experimentally soon after the invention of the laser. In particular, the first experimental observation of two-photon excitation was in 1961.^11^

It is often stated that two-photon absorption is much weaker than single-photon absorption because the cross-section is much smaller. But, although it is indeed much weaker, as two photons must be in the same vicinity at almost the same time, the cross-sections cannot be directly compared as they have different units (${\mathrm{cm}}^{4}\;s$ as compared with ${\mathrm{cm}}^{2}$). As a consequence, if the unit of distance was taken as an angstrom, for example, the cross-sections would not be so different in magnitude.

Beer’s law, for the depletion of a beam intensity with distance x, is modified for two-photon absorption.^12^ It becomes, rather than an exponential decay,

$$I=\frac{I_{0}}{1+\beta xI_{0}},$$ (1)

where $I_{0}$ is the original intensity and β is the two-photon absorption coefficient.

## 2 Nonlinear and Multiphoton Microscopy

The first published nonlinear microscope images formed in a scanning mode, from second harmonic generation (SHG), were presented as a post-deadline paper at a conference in 1977^13^ and subsequently in a paper in 1978.^14^ Our group in Oxford was led by Rudi Kompfner, who had already published images from SHG in the scanning acoustic microscope:^15^

“At the focus the acoustic beam converges to a diameter comparable with the acoustic wavelength. Because of this sharp convergence the intensity at the focus can be large. It, therefore, occurred to us to look for the nonlinear effects which become pronounced at high intensities.”

In another paper published by our group in 1978, the advantage of scanning using a highly focused laser beam for the production of nonlinear optical effects, in general, was highlighted as follows:^16^

“In the scanning optical microscope nonlinear interactions are expected to occur between the object and a highly focused beam of light, which we hope will open new ways of studying matter in microscopic detail hitherto not available. Nonlinear interactions include the generation of sum frequencies, Raman scattering, two-photon fluorescence, and others. We feel that the method will be of particular interest in studying biological materials, some of which have large second-harmonic generation coefficients, and the wide range of these coefficients should give very strong contrast in the images formed. Furthermore, frequency mixing should give information concerning the chemical structure of the object.”

In particular, we were actively working on the experimental demonstration of SHG imaging, but in addition, specifically proposed other nonlinear imaging modes, including two-photon fluorescence and coherent Raman scattering.

**Fig. 1:**
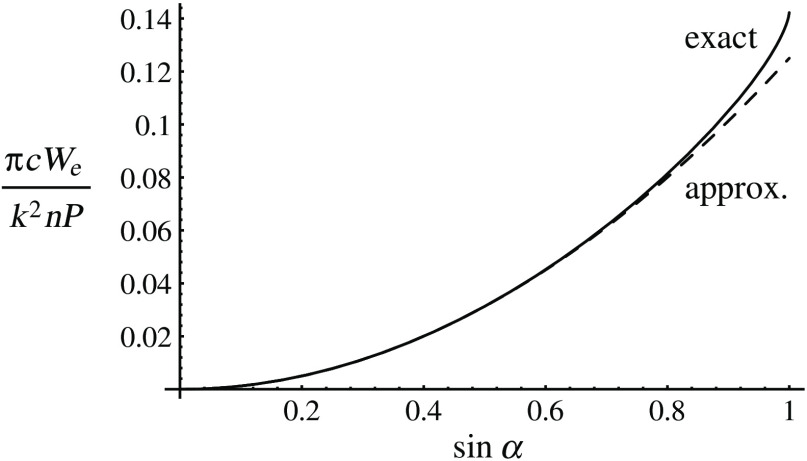
The electric energy density at the focus of a high numerical aperture microscope objective.

The normalized electric energy density $W_{e}$ at the focus of a high numerical aperture aplanatic microscope objective for an instantaneous power P, calculated using the theory of Richards,^17^ is

$$W_{e}=\frac{k^{2}nP}{\pi c}\frac{{\left[5(1-\cos^{3/2}\;\alpha )+3(1-\cos^{5/2}\;\alpha )\right]}^{2}}{450\;\sin^{2}\;\alpha },$$ (2)

where n sin α is the numerical aperture and k=2π/λ, as shown in Fig. 1.^18^^,^^19^ The dashed curve gives the approximate prediction of a scalar, paraxial approximation.

Our SHG images were acquired using a continuous-wave YAG laser, although we knew the advantages of using a short-pulsed laser and also experimented with a mode-locked (ps) argon ion laser. We looked at various inorganic crystals such as KD*P, ${\mathrm{LiNbO}}_{3}$, ZnO, and quartz. We demonstrated that SHG imaging exhibits an optical sectioning property without the use of a confocal pinhole:^14^

“Detail outside the focal plane does not interfere with the image as much as in a conventional microscope since the harmonic generated is proportional to the intensity squared and this results in the main contribution only coming from the region of focus where the intensity is very large.”

This optical sectioning property allows three-dimensional imaging of thick samples, such as tissue. It is also exhibited by two-photon fluorescence microscopy (2PFM) and is responsible, together with the reduced scattering of the longer wavelength excitation, to the improved penetration depth of two-photon fluorescence or SHG microscopy.

The SHG approach was extended to biological samples by Freund in 1986.^20^ The first reported coherent anti-Stokes Raman spectroscopy microscope image was by Duncan et al. in 1982.^21^

The first published report of 2PFM was by Denk et al. in 1990.^22^ They stressed that photobleaching (from two-photon absorption) is much reduced as compared with single-photon fluorescence microscopy, as a result of the optical sectioning property.

Three-photon fluorescence microscopy was reported in 1996.^23^^,^^24^ SHG microscopy using femtosecond pulses, and combined SHG and 2PFM, was reported by Gauderon et al.^25^^,^^26^ Third-harmonic generation (THG) microscopy was reported by Barad et al.^27^

Image formation and resolution in two-photon and three-photon microscopy have been investigated in various papers.^28^^–^^34^ Although combining two-photon microscopy with a confocal pinhole gives an improved spatial resolution and optical sectioning,^28^^,^^31^^,^^33^ usually signal is low, so that as much light as possible must be detected. For this reason, nondescanned detection is often used to increase detection efficiency.^35^ However, there has been some experimental evidence that signal-to-noise-ratio is optimized for a particular pinhole size.^34^^,^^36^ Some researchers seem to think that 2PFM gives better resolution than confocal microscopy, but this is not the case when the same emission wavelength is assumed for each case.^28^ Partially coherent image formation in SHG microscopy has also been studied.^37^ Although the SHG process is itself coherent, integration over a finite-sized detector gives an overall partially coherent effect.

An important pulse-shaping technique that has been applied to multiphoton microscopy is temporal focusing.^38^^,^^39^ The different spectral components of the laser pulse are spread out and recombined in the focus. Then at regions far from focus, the pulse length at any point is long, so that multiphoton signal is reduced. Temporal focusing gives an optical sectioning effect, even for line-scan or widefield excitation.

An analysis of two-photon imaging in scattering media, such as biological tissue, has shown that the fundamental limitation to penetration depth is determined by background that emanates from the sample surface.^40^^,^^41^ The mechanism is that, as the excitation within the sample is reduced by scattering, and as two-photon absorption is a square law process, the two-photon fluorescence signal is eventually reduced below the background level.

## 3 Pulse Length

Because of the nonlinearity (square-law behavior) of two-photon processes, for either two-photon fluorescence or SHG, short pulses should be used to maximize the two-photon signal. This was appreciated and analyzed in a report written by our group in 1975 (available on ResearchGate),^42^

“Then it can be shown that the shorter the pulses can be made, keeping the mean power constant, the higher will be the instantaneous intensity, and hence the greater will be the harmonic signal produced. It is therefore desirable to use a light source capable of producing the shortest possible pulses, consistent with adequate mean power i.e., Neodymium-YAG lasers, or dye-lasers.”

This was, of course, before the invention of the titanium sapphire laser. It was also mentioned in our paper:^14^

“It is possible to increase the harmonic signal for a given specimen temperature rise by pulsing the laser. Fast-scanning of the beam results in the whole specimen reaching an equilibrium temperature, in contrast to the slow-scan case where the probe is effectively stationary on the object, and allows a higher incident power.”

The report also discusses confocal microscopy and scanning near-field microscopy, extracts on which were reprinted later.^43^ In particular, experimental results were obtained on superesolution using a saturable absorber.

Actually, laser pulses can be too short for efficient two-photon absorption. If the frequency spread of the laser pulses is greater than the bandwidth of the absorption spectrum of the sample, absorption will be reduced.

Denk et al. filed a patent for two-photon microscopy using pulses shorter than 1 ps,^44^ because, as we have discussed, 2PFM itself had been proposed many years earlier. Although short pulses increase the two-photon signal, the specification of 1 ps seems rather arbitrary. Hanninen and Hell^45^ subsequently patented multiphoton microscopy using pulses greater than 1 ps. These patents have now expired, and several companies manufacture multiphoton microscopes.

A laser pulse of peak power P and duration t results in a two-photon energy signal

$$E_{2-p}=aP^{2}t,$$ (3)

where a is a constant that depends on the two-photon cross-section. It is also well known (e.g., from Refs. 14, 16, and 42) that the average power that can be focused on to the sample is limited because of heating and other undesirable side effects. The average power is given as

$$P_{\mathrm{av}}=Pt/T,$$ (4)

where T is the time interval between pulses. We have during the pulse

$$E_{2-p}=aP_{\mathrm{av}}^{2}T^{2}/t,$$ (5)

so that the average two-photon signal power is given as

$$P_{2-p}=aP_{\mathrm{av}}^{2}T/t,$$ (6)

and thus the signal is increased as the pulse length decreases while the average power is held constant.

However, an ultimate limit to how short the pulses can be is set by the onset of optical breakdown. Docchio et al.^46^ showed that the power density needed to cause optical breakdown in water actually increases as the pulse length is decreased. Stern et al.^47^ showed that the power needed to cause surface ablation of the cornea can be expressed as

$$P=bA/t^{1/2},$$ (7)

where b is a constant and A is the cross-sectional area of the focused spot. At breakdown, we would thus expect a two-photon signal

$$P_{2-p}=ab^{2}A^{2}/T.$$ (8)

Thus, it is seen that in this case the two-photon signal at the onset of breakdown is independent of the pulse length t.

In order to obtain the maximum two-photon signal for a given average power, the pulse length can thus be decreased until close to breakdown. Breakdown occurs when

$$t=(P_{\mathrm{av}}T/bA),$$ (9)

which is thus independent of a, and therefore of the two-photon cross-section. Thus, one does not need to know the two-photon cross-section in order to predict the minimum pulse length that can be used to maximize the two-photon signal while avoiding breakdown. Taking typical values of breakdown given by Docchio et al.,^46^ and Stern et al.^47^ give a value of b of about $4\times 10^{6}\;{\mathrm{Js}}^{-1/2}\;{\mathrm{cm}}^{-2}$. For ordinary mode-locked lasers, T is about $1.25\times 10^{-8}\;s$. We find that for an average power of 100 mW and $A=2\times 10^{-9}\;{\mathrm{cm}}^{-2}$, t can be reduced to about 20 fs while still avoiding breakdown. For an average power of 10 mW, t is less than 1 fs for breakdown to occur. Thus, onset of breakdown occurs at subpicosecond pulse lengths for any reasonable value of average power.

**Fig. 2:**
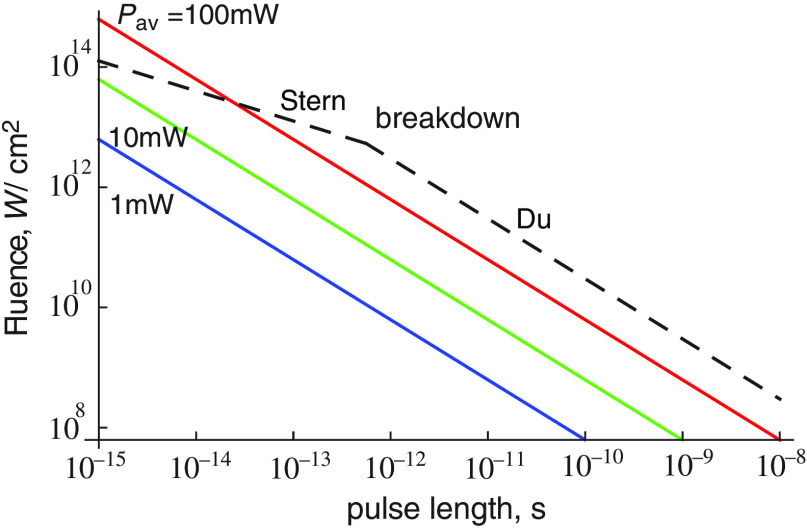
Beam fluence as a function of pulse length for a given average power. T is taken as $1.25\times 10^{-8}\;s$. A is taken as $2\times 10^{-9}\;{\mathrm{cm}}^{-2}$. The onset of breakdown is also indicated.

However, recent results show that even these figures for breakdown are lower than those found for subpicosecond pulses. From Du et al.^48^ and Tien et al.^49^ for fused silica, the damage threshold can conservatively be taken to be constant at about $3\;J/{\mathrm{cm}}^{2}$ for pulses shorter than about 10 ps. Thus for subpicosecond pulses, the fluence at breakdown is inversely proportional to the pulse length. The result is that pulses of average power <100  mW breakdown never occurs for any pulse length. These predictions, together with the behavior of the fluence for constant average power, are shown in Fig. 2.

In addition, Docchio et al.^46^ also showed that the power density needed to cause breakdown increases as the size of the focused spot decreases. So for a high numerical aperture lens, one would expect that even shorter pulses could be used without causing breakdown. Although the data in Docchio et al.^46^ do not allow an accurate extrapolation of the effect of increasing the numerical aperture to the levels used in scanning laser microscopy, we can estimate that, for the parameters given above, average powers in the order of watts would be needed to cause breakdown.

The fluence for a given two-photon power can also be calculated from Eq. (3) or Eq. (6). We obtain

$$F=\frac{1}{A}{\left(\frac{P_{2-p}T}{at}\right)}^{1/2},$$ (10)

so that lines of constant two-photon signal for pulses longer than about a picosecond are parallel to the breakdown curve in Fig. 2. Thus in this regime, the two-photon signal at the onset of breakdown is independent of pulse length.

In the above, we have assumed a repetition rate $T^{-1}$ appropriate for an ordinary mode-locked laser. Then many pulses will arrive during the pixel dwell time, so T can be increased. From Eq. (6), the average two-photon signal power is then also increased. In fact, it can be increased so much that wide-field imaging becomes feasible.^50^^,^^51^ The optimum repetition rate for two-photon excitation has been investigated, but the interaction of many different effects complicates the overall behavior.^52^

## 4 Contrast Mechanisms

Multiphoton fluorescence microscopy can be performed using autofluorescence or by labeling with dyes. An alternative for confocal microscopy is to use conjugated nanoparticles, such as 5- to 15-nm gold nanospheres.^53^ Nanoparticles, such as nanospheres, nanoshells, and nanorods, avoid photobleaching of fluorescent labels.

SHG occurs from noncentrosymmetric structures.^54^ These include as examples collagen and actin. Polarized SHG can be used to determine the nonlinear permittivity elements.^55^^,^^56^ Illumination using radially polarized light produces a longitudinal electric field, allowing observation and determination of additional elements.^57^ The surface of a centrosymmetric crystal can also generate a harmonic signal.^58^^,^^59^ Plasmonic enhancement from a rough surface of $\times 10^{4}$ has been reported.^60^ In a centrosymmetric metallic medium, the second-order polarization $P^{\left(2\right)}$ is of the form^61^

$$P^{\left(2\right)}=\alpha (E\cdot \nabla )E+\beta E\cdot \nabla E+\gamma E\times (\nabla \times E),$$ (11)

where α and β (electric quadrupole term) are constants giving the strength of surface terms, and γ gives the strength of a magnetic dipole, volume term. A theory for SHG from a spherical particle was developed.^62^ It was shown that SHG can also give rise to enhanced backscattering from rough surfaces (a peak in scattering in the reverse direction of the incident beam).^63^ Surface molecular monolayers have also been detected by SHG.^64^

SHG is a coherent signal, but an incoherent signal, hyper-Rayleigh scattering (HRS), was observed from colloids.^65^^,^^66^ Nappa et al.^67^ observed that SHG from small particles was dipolar in nature, but from larger particles, >80  nm, quadrupole scattering was observed. HRS has been observed from nanorods.^68^ SHG has been observed from gold nanospheres conjugated to antibodies,^69^ and SHG microscopy was performed with fluorescent polymer encapsulated gold nanoparticles.^70^

Two-photon luminescence (TPL), an “unusual broad luminescence background,” was observed from noble metals and enhanced by roughened surfaces.^60^^,^^71^ This led to plasmon-enhanced TPL microscopy^72^ and *in vivo* TPL microscopy using gold nanorods.^36^ Usually, the nanorods are excited at resonance, but Balla et al. proposed excitation of nanorods off-resonance (at 1200 nm). While the luminescence signal is reduced, this has the advantages of increased excitation wavelength and therefore penetration, allowing signal detection in the near-infrared, and of reduced heating of the nanoparticles.^73^ THG gives contrast from interfaces.^27^ THG from single gold nanoparticles has also been observed.^74^

## 5 Two-Photon Focal Modulation Microscopy

We finish with two recently developed techniques that can be applied to multiphoton microscopy. The first is focal modulation microscopy (FMM).^75^ In this technique, a laser beam is divided into two beams one of which is frequency-shifted. These two beams illuminate different regions of the illuminating lens pupil, a variety of different geometries having been proposed. The beams intersect in the focal region, producing an intensity modulated at the difference frequency. The technique thus has strong similarities with temporal focusing. The generated fluorescence signal is also modulated and is detected using lock-in techniques. The result is better penetration into a scattering medium and an improved spatial resolution.^76^ This method can also be applied to 2PFM.^77^^,^^78^ Compared with conventional two-photon microscopy, transverse resolution is increased by 70%, while the axial resolution is increased twofold. Results showed that the signal-to-background ratio of 2PF-FMM can be up to five times better than in regular two-photon microscopy at the depth of 500  μm.^78^

## 6 Two-Photon Image Scanning Microscopy

Another promising new microscopical technique is image scanning microscopy (ISM).^79^^,^^80^ In this approach, the pinhole of a confocal microscope is replaced by a detector array. The signals from the detector elements are reassigned to the correct image location, which is to a first approximation for single-photon fluorescence midway between the illumination and detection points, and then summed.^79^^–^^81^ The result is a combination of improved transverse and axial resolution and an improved signal-to-noise ratio. The detected power is determined by the size of the detector array. Transverse resolution for an offset detector pixel has been found to be improved relative to the central pixel,^82^ so overall transverse resolution for the detector array can be even better than for confocal microscopy with a small pinhole. Our implementation uses an array of 5×5 avalanche photodiodes.^83^

ISM can also be successfully applied to multiphoton microscopy.^84^^–^^86^ This improves the optical sectioning property of two-photon microscopy, as a result of an additional confocal sectioning effect. Although a confocal pinhole is rarely used for two-photon microscopy, the penetration depth for ISM can be increased because the confocal sectioning effect reduces the strength of the background from the sample surface.^40^^,^^41^ A theoretical treatment of two-photon ISM has also been presented.^87^

## 7 Conclusion

We have discussed the historical development of multiphoton microscopy and some of its major features. We have also indicated two promising future developments for multiphoton microscopy, namely FMM and ISM. Much more detail can be found in the excellent books edited by Alberto Diaspro and Karsten König and the reprint collection of Barry Masters.^88^^–^^90^
